# Electro-polymerization of modified carbon paste sensor for detecting azithromycin

**DOI:** 10.1038/s41598-024-79614-6

**Published:** 2025-01-06

**Authors:** Salma Mamdouh, M. Shehata, A. M. Fekry, M. A. Ameer

**Affiliations:** Chemistry Department, Faculty of Science, Cairo University, Giza, 12613 Egypt

**Keywords:** L-Threonine, COVID-19, Electro-polymerization, Antiviral, Azithromycin, Environmental sciences, Chemistry

## Abstract

Azithromycin (AM) is one of the prescribed drugs in pandemic medication treatment which has paid great attention. We developed in this study a simply modified carbon paste electrode (CPE) to detect AM using poly-threonine (PT). PT or similar polymers are used as carriers to enhance the delivery and effectiveness of AM. The work was characterised via Cyclic Voltammetry (CV), electrochemical impedance spectroscopy (EIS) and scanning electron microscopy (SEM). We take into consideration the effects of pH, scan rate, accumulation time, interference, and calibration curve.A very sensitive response to the oxidation of 1.0 mM from AM in phosphate buffer solution (PBS) over a pH range of 5.0 to 10.0 was observed using the developed poly-threonine carbon paste electrode (PTCPE). The impact of different AM concentrations was investigated resulting in a detection limit of 0.32 µM and a quantification limit of 1.07 µM at PBS (pH 7.4). Finally, the recently used electrode realized acceptable sensitivity and consistency for AM detection in pharmaceutical drugs.

**Supplementary Information:**

The online version contains supplementary material available at 10.1038/s41598-024-79614-6.

## Introduction

The semisynthetic macrolide antibiotic AM, (9-Deoxo-9a-aza-9a-methyl-9a-homoerythromycin A) (Supplementary Fig. S1A) was generated by remodeling the naturally occurring antibiotic, erythromycin (Supplementary Fig. S1B) through replacing the carbonyl group at the 9a site of the aglycone ring by a methyl-substituted nitrogen, resulting in a 15-membered ring termed as an azalide^1^. AM exhibits a broad spectrum of antibacterial activity, as a result, it is in use typically to treat a wide range of mild-to-moderate bacterial infections. As well as its antibacterial effects, AM has been reported to have antiviral properties in vitro and/or in vivo against a variety of viruses, including Ebola^2^, Zika^3^ and influenza H1N1 virus^4^.

AM also displayed antiviral activity against SARS-CoV-2^5–9^. The world health organization (WHO) described COVID-19 as a global pandemic that has produced millions of fatalities globally. AM detection in real samples is important as being one of the prescribed medications alongside paracetamol, zinc and vitamin C in the treatment regimen in many countries including Egypt^10^. AM has the potential to cause serious adverse effects. Headache, nausea, dizziness, stomach aches and hepatotoxicity were the most commonly reported side effects^8^. Also, according to a preceding study, AM is likely to cause abnormal cardiac activity^11^. Long-term usage of AM may result in antibiotic resistance and even mortality in individuals who suffer from allergy to it^12^. Different analytical and electrochemical methodologies for detecting AM were presented in several studies. For instance; Raghuram et al. used reversed-phase high performance liquid chromatography (RP-HPLC)^13^, El-Adl et al. employed p-chloranilic acid in their spectrophotometric analysis^14^, Chavada et al. utilized colorimetric sensing^15^ and Guo et al. applied a fluorescent probe using nitrogen and sulfur co-doped carbon quantum dots^16^. These analytical approaches are unsatisfactory since they are time-consuming, want an expert for complex devices, incur excessive costs and demand preparation of the drug prior to analysis.

However, electrochemical techniques were a superior alternative for these restrictions since they offer a simple electrode preparation, high sensitivity, prompt response, and are affordable and timesaving^17–23^. For example; Stoian et al., Rebelo et al., Zhou et al. and Jafari et al. utilized molecularly imprinted polymers (MIPs)^24–27^, Vajdle et al. employed a silver-amalgam film electrode^28^, Sharma et al. used zinc vanadate/phosphorous doped reduced graphene oxide nanostructure^29^. These adjustments, however, produced quite reasonable results, but they have some faults, such as the use of exorbitant raw materials, complex construction of the working electrodes and engaging with hazardous metals.

The choice to use CPE was rather satisfactory because it has several advantages as being affordable, easy to handle, safe and having minimal background currents over a wide array of potentials^30–34^.

This study offers a new CPE modification^35–39^ that uses PT to detect AM. Threonine, (Supplementary Fig. S1C) is a key component of numerous body proteins and is an essential amino acid. It is an immune-boosting nutrient along with serine and is one of the two proteinogenic amino acids with an alcohol group. Along with isoleucine, it is one of the two common amino acids containing a chiral side chain^40^. Electro-polymerization has emerged as an effective and adaptable methodology for manufacturing customized electrodes. The application of thick polymer coatings provides an extremely high surface coverage, which gives this modification a lot of versatility. The surface of the PTCPE was analyzed using SEM and EIS. Various voltammetric techniques were used to test the feasibility of employing the PTCPE sensor to detect AM.

This work aims to create a new simple PTCP sensor for AM detection which has proved its efficacy as being a sensitive, selective and low cost fabricated electrode. In addition, the utility of the proposed sensor was examined in real pharmaceutical samples and showed effective response toward AM.

## Experimental

### Chemicals

AM in its pure state and methanol (for HPLC ≥ 99.9%) were purchased from Sigma-Aldrich to prepare a 10.0  mM AM stock solution. potassium ferrocyanide, potassium chloride and graphite micro-particles (< 50.0 μm, for the CPE preparation) were obtained from Merck, Darmstadt, Germany. Paraffin oil was bought from Aldrich, USA. Sodium hydroxide was obtained from the International Trade Association (ITA). Sodium dihydrogen orthophosphate and disodium hydrogen orthophosphate were obtained from El Nasr Pharmaceutical Chemicals Co. (ADWIC) for the preparation of 0.1 M, pH 7.0 PBS. L-Threonine (> 99.0–101.0%) was purchased from Bio Basic Inc.

AM’s medicinal samples (Zithrokan capsules, Hikma pharmaceuticals, Cairo, Egypt) were purchased from a local pharmacy. Many pharmaceutical medications were employed in the interference test, including Cephalexin (Ceporex tablets, GlaxoSmithKline (GSK), Cairo, Egypt), Clarithromycin (KLACID XL tablets, Abbott, Cairo, Egypt) and Paracetamol (Sigma-Aldrich). Urea, starch, sucrose and glucose were bought from MISR-Scientific Company.

### Apparatuses and methods

The electrochemical studies were conducted at room temperature utilizing a standard 25.0 mL three-electrode setup. The working electrode (WE) was either the bare electrode or the PTCPE, while a saturated calomel electrode served as the reference electrode (RE), and a platinum rod functioned as the counter electrode (CE).

The electrochemical cell was linked to a computer-operated EC-Lab^®^ electrochemical software and a Bio-logic SAS model SP-150 potentiostat. All electrochemical assays, including CV, SWV and EIS, were carried out under the same circumstances as in our earlier study^41^. Using sinusoidal voltage amplitude of 10.0 mV, EIS measurements was performed between 100.0 mHz and 100.0 kHz in the frequency range. The EC-Lab^®^ program was utilized to perform the fitting and analysis of the data, employing the most optimal equivalent circuit model. Measurements were done at least three times to achieve repeatable results.

The Adwa 1030 digital pH meter (Romania) was connected to measure the pH solution. SEM (Model Quanta 250 Field Emission Gun) was used to examine the morphology (FEI Company, Japan).

### PTCPE sensor fabrication

At first, carbon paste (CP) was prepared by hand mingling of 3.0 g graphite powder with an appropriate amount of paraffin oil in a mortar for 10.0 min to get a consistent paste. Then, to obtain a CPE, this paste was inserted into a 3.0 mm diameter hole at the tip of a Teflon tube. Subsequently, the electrode’s surface was then pressed utilizing sandpaper.

Eventually, the polymeric layer of PT formed on the surface of the CP was attained by means of a CV in the potential range of − 0.6 to 2.3 V with a scan rate of 0.1 V/s for 30.0 scan cycles at PBS of pH 9.0 in the existence of L-threonine with a concentration equals 2.5 mM. After that, the resultant adapted electrode was washed with distilled water and then air-dried to be available for assessing AM^42^.

### Pharmaceutical samples preparation

One AM capsule with 500.0 mg dosage was emptied into 250.0 mL distilled water and stirred magnetically for 30.0 min, let to settle down, and decanted. Finally, 2.5 mL of this solution was added to 22.5 mL PBS in the electrochemical cell to measure the contents of AM through the standard addition method as previously stated in our work^43^.

## Results and discussion

### Electro-polymerization of L-Threonine on the surface of CPE

**Fig. 1:**
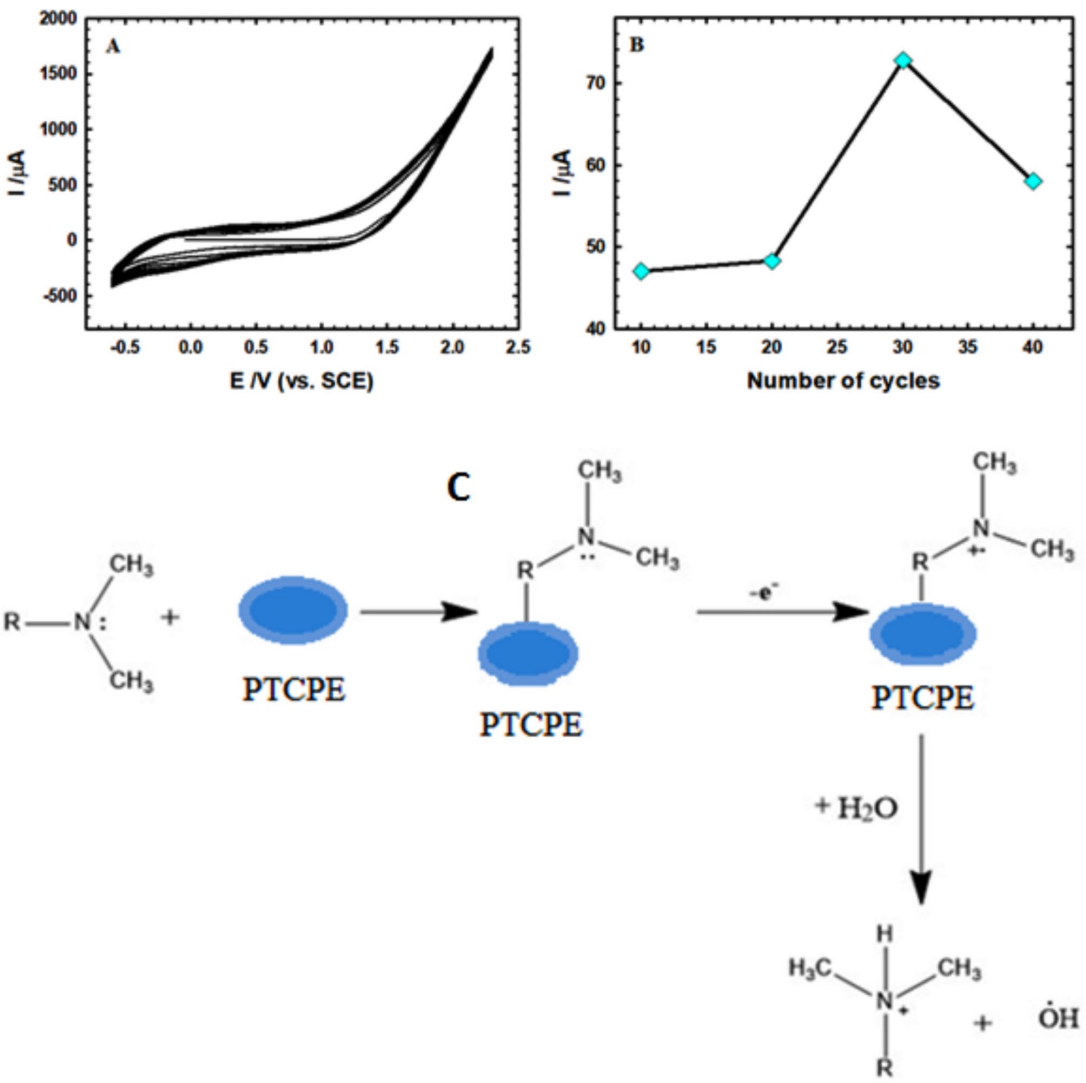
(**A**) CVs for the electro-polymerization of L-threonine (2.5 mM) on CPE using potential range of − 0.6 to 2.3 V with a scan rate of 0.1 V/s for 30.0 scan cycles at 0.1 M PBS (pH 9.0). (**B**) Effect of the number of cycles (10–40 cycles) on the anodic current. (**C**) The proposed electro-oxidation mechanism of AM on PTCPE.

The outcome of the number of electro-polymerization cycles was inspected by scanning the electrode within a potential range of − 0.6 to 2.3 V at a scan rate of 0.1 V/s for 30.0 scan cycles in PBS of pH 9.0 as revealed in Fig. 1A. The variation of the peak current versus the number of scan cycles is shown in Fig. 1B. The current values amplified with increasing the number of cycles from 10.0 to 30.0 and then decayed. The uppermost was obtained for 30.0 scan cycles, so it was employed for the electro-polymerization of L-Threonine on the CPE surface.

### Surface characterization

The surface morphology of PTCPE is demonstrated in Supplementary Fig. S1D with a cotton needles like surface with a uniform arrangement confirming the successful electro-polymerization of L-Threonine on the surface of the CPE.

### Electrochemical functioning of AM at the surface of the PTCPE

**Fig. 2:**
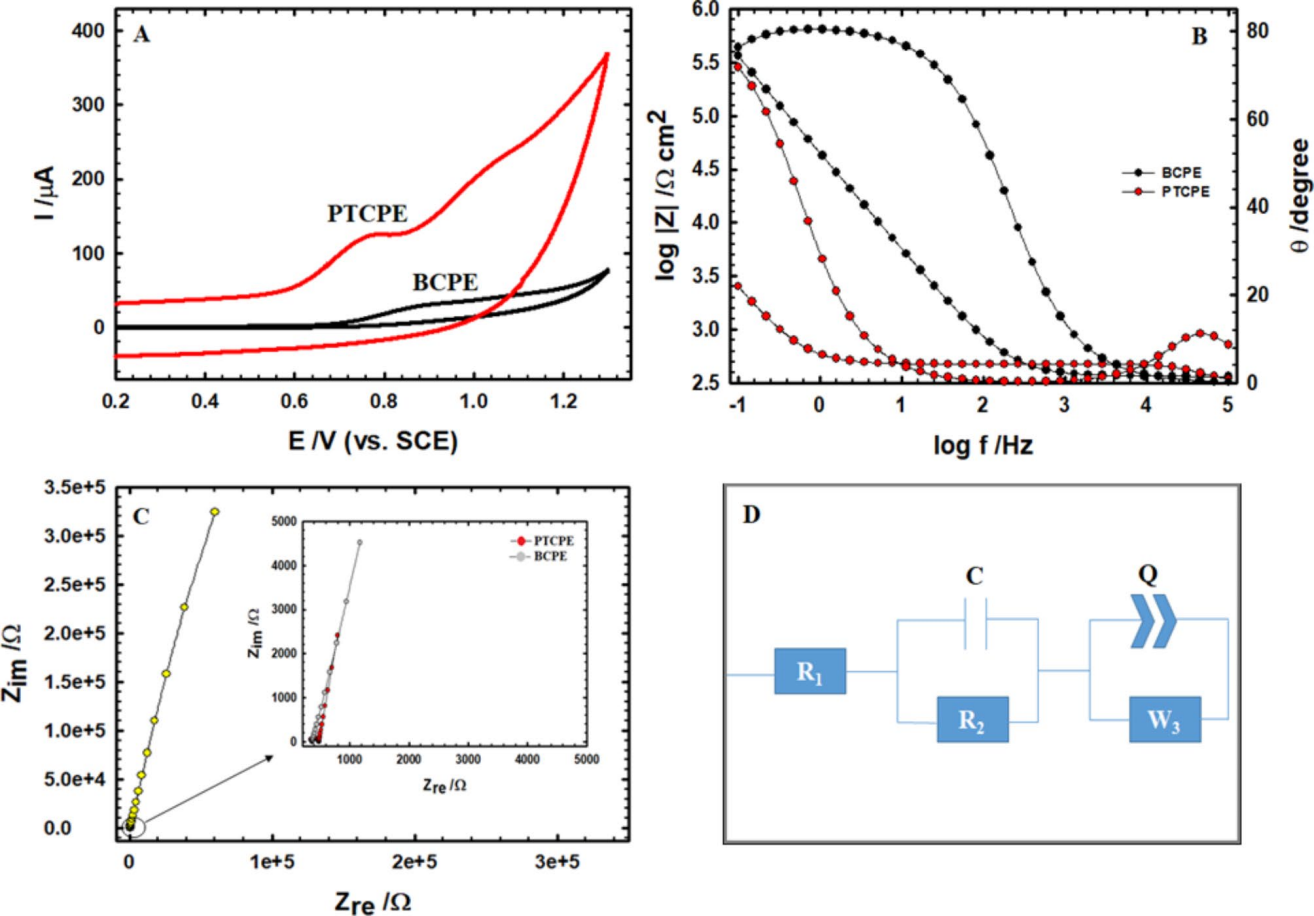
(**A**) AM CVs in pH 7.4 PBS (0.1 M) with a scan rate of 0.05 V/s at BCPE and PTCPE. (**B**,**C**) EIS analysis at AM (1.0 mM) in pH 7.4 PBS (0.1 M) (Bode and Nyquist plots, respectively). (**C** inset: Nyquist plot with high amplification). (**D**) The equivalent circuit with the best performance.

To optimize any electrochemical sensor, it is vital to understand how it reacts with AM when compared to BCPE. The CV technique was used to test the sensitivity and validity of employing the PTCPE to detect AM. At a scan rate of 0.05 V/s, the CVs of BCPE and PTCPE in PBS (0.1 M, pH 7.4) with 1.0 mM AM are shown in Fig. 2A. The oxidation of AM on PTCPE appears to be an irreversible process with two distinct oxidation peaks, the first at around 0.8 V with peak current around 90.0 µA, which is 4.5 times larger than that of the bare electrode (20.0 µA), while the second happens at a more positive potential of around 1.0 V.

These peaks can be justified by the fact that the AM molecule is adsorbed on the surface of PTCPE through different pathways. PT may act as carriers to enhance the delivery and effectiveness of AM which contains several functional groups that can participate in hydrogen bonding, such as hydroxyl (–OH) and amine (–NH) groups. PT features hydroxyl groups due to the presence of threonine’s side chain, potentially allowing for hydrogen bonding interactions with AM and strong attaching with it. Also PT is polar polymer due to its hydroxyl groups; there might be chance for the polymer that can interact hydrophobically with hydrophobic regions of AM. Such interactions would be stronger in aqueous environments as the case in our investigation where they can reorganize each other.

Through the last mentioned interaction, a certain electro-oxidation by losing an electron from the nitrogen atom in the desosamine sugar residue, forming a radical cation, and then protonation of this nitrogen by drawing a proton from the water residue in the media. Because this final state is electro-inactive, no more oxidation happens (Fig. 1C). According to the theory that the electro-oxidation of AM is followed by a quick chemical reaction and a second electron-transfer step (ECE mechanism), a second peak arises at a greater positive potential, due to the oxidation of the chemical reaction’s byproducts^44^.

EIS analyses, which encompass both Bode and Nyquist plots (Fig. 2B,C, respectively), are a convincing tool for approving the CV data and ensuring that PTCPE has a higher electro-catalytic activity than BCPE for the oxidation of 1.0 mM AM. The Nyquist plot for BCPE is a straight line while that for PTCPE is mainly a straight line with an insignificant semi-circle part signifying that the development is primarily diffusion-dependent with slight charge-transfer reliance. The data are clearly illustrated by the model shown in Fig. 2D with a moderate error of 1.0%. Where; R1 reflects the solution’s resistance, C is the double layer capacitance, which is linked in parallel to R2 “the outer layers’ resistance”, Q is the constant phase element which is also linked in parallel to W “the Warburg component related to the diffusion process”. An empirical coefficient (α = 0.0 to 1.0) explains the deviation from the capacitive ideality as a consequence of surface roughness, α = 1.0 stands for an ideal capacitor and α = 0.5 stands for a diffusion behavior^45^. For the two electrodes, PTCPE and BCPE, The best-fitting values are listed in Table 1. The value of R1 is relatively constant for each electrode. The greater capacitance values and lower impedance values of PTCPE when compared to BCPE imply a stronger conducting performance and guarantee the high oxidation peak current found in the CV data.

**Table 1:** EIS data of AM (1.0 mM) in 0.1 M PBS at the surface of PTCPE and BCPE.

R_1_ + (C/R_2_) + (Q/W)	Value	
	PTCPE	BCPE
R_1_ (kΩ)	0.32	0.36
C (µF)	260.00	7.46
R_2_ (kΩ)	0.156	2660
Q (µF)	629.50	10.24
W (MΩ/s^1/2^)	272.0	376.0

### Influence of solution pH

**Fig. 3:**
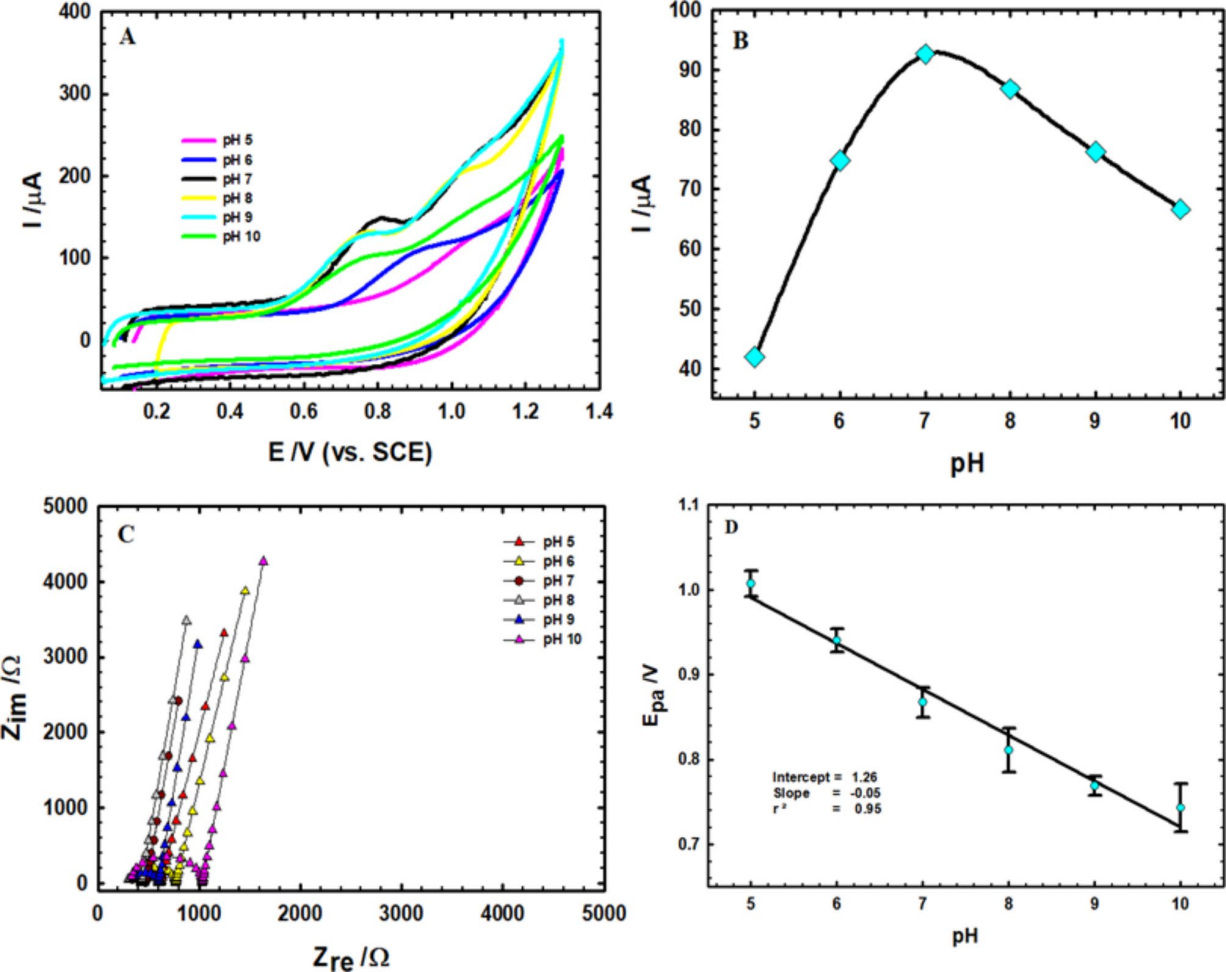
(**A**) CVs of AM (1.0 mM) in PBS 0.1 M at varying pH values (5.0–10.0) scanned at 0.05 V/s. (**B**) The anodic current related to the pH for PTCPE. (**C**) Nyquist plots of 1.0 mM AM at varying pH values. (**D**) The effect of pH on the anodic potential of AM at PTCPE.

The effect of adjusting pH on the electro-oxidation of 1.0 mM of AM in 0.1 M PBS (pH 5.0–10.0) at PTCPE was exhibited using the CV technique (Fig. 3A). The electro-oxidation of AM is clearly pH-dependent as the current peak widens and begins to disappear in acidic medium (pH 5.0), which is due to the AM molecule being protonated and the fact that AM is only electro-active when it is not protonated. When the PT chain is ionized depending on the pH, it may carry a positive or negative charge that makes electrostatic attractions with AM, which may also hold ionizable regions in the operated pH of the study.

As the pH rises, an ideal peak emerges. For this work, pH 7.4 was applied to imitate the physiological pH of the human body.

The current surges on going from pH 5.0 to 8.0, according to the influence of pH on the current peak (Fig. 3B), and subsequently collapses at higher pH values, confirming the mechanism of AM protonation in acidic medium while remaining electro-inactive. Because AM has a pKa value of 8.7, it is understandable why the present peak values for the pH range 7.0–8.0 are relatively high.

Nyquist plot (Fig. 3C) conforms to the CV’s outcomes for changing the pH of PBS. It shows that pH 7.4 acquires the lowermost impedance value with the highest conductivity.

The relationship between pH and peak potential is shown to be linear in Fig. 3D, providing the following equation: E_p_ (V) = 1.26 − 0.05 pH (r^2^ = 0.95).

This demonstrates that the electro-oxidation of AM requires a proton-transfer phase and that the anodic peak potential is dependent on pH. The slope (0.050 V/pH) matches the ideal nernestian slope at 25 °C (0.059 V/pH), proving that the same number of protons and electrons were exchanged during the electrochemical oxidation.

### Influence of scan rate

**Fig. 4:**
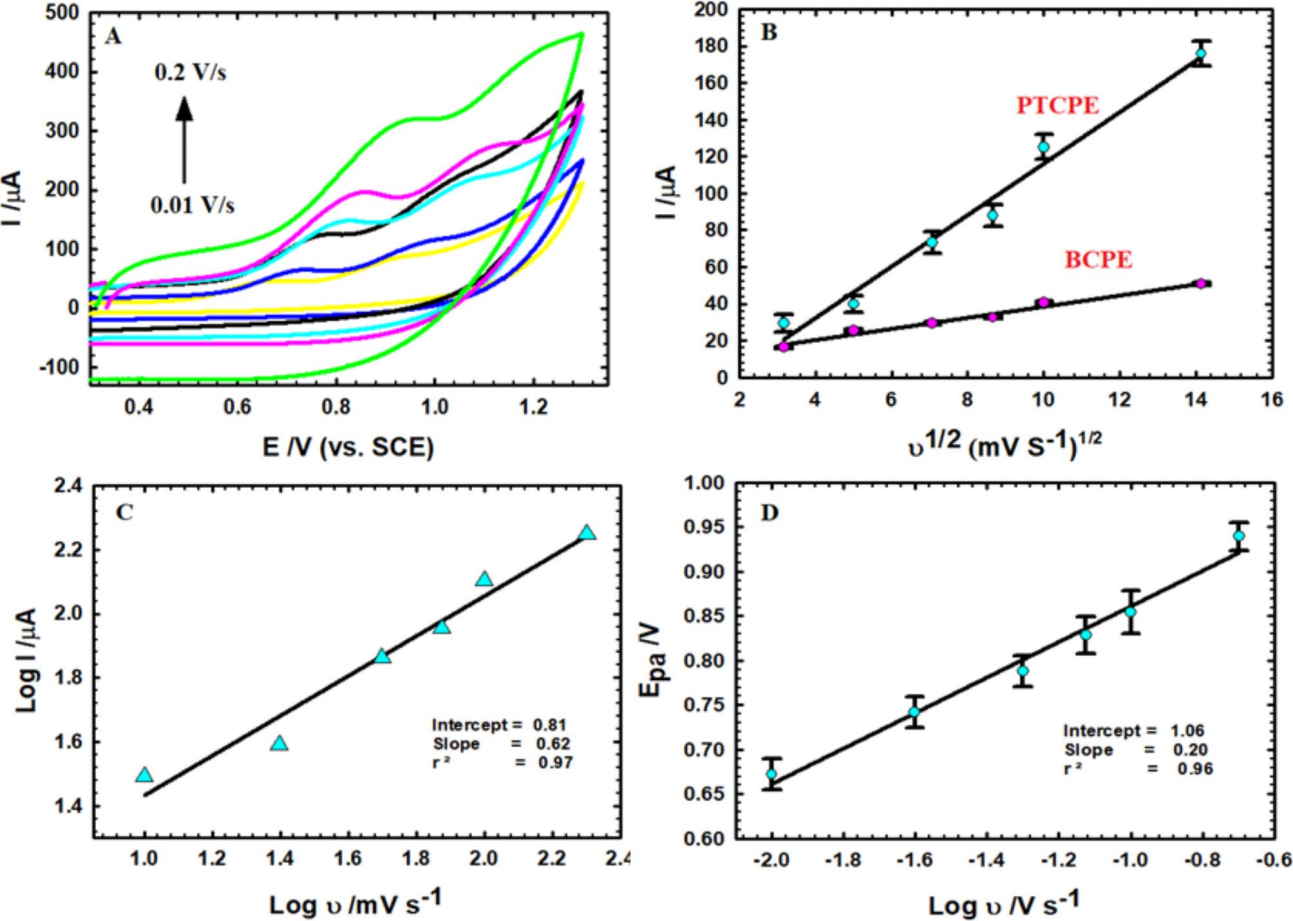
(**A**) CVs of AM (1.0 mM) in pH 7.4 PBS at varying scan rates (0.01–0.2 V/s). (**B**) The anodic peak current versus the square root of scan rate at BCPE and PTCPE. (**C**) The logarithm of the anodic current related to the logarithm of the scan rate at PTCPE. (**D**) The anodic potentials plotted versus the logarithm of the scan rate at PTCPE.

The CV approach in Fig. 4A illustrates how the scan rate affects the anodic peak current of AM with 1.0 mM concentration in PBS (0.1 M). The peak current escalates and the peak potential shifts to higher affirmative values as the scan rate upsurges from 0.01 to 0.20 V/s, viewing an irreversible electrochemical oxidation.

According to Fig. 4B, there is linearity amongst the anodic peak current and the square root of the scan rate, declaring the existence of a diffusion-controlled mechanism, this linear relation is illustrated by:

$$\begin{array}{*{20}l} {\text{I}}\left( {\upmu {\text{A}}} \right) = 3.05\upupsilon ^{1/2} + 8.02; \hfill &\quad {\text{r}}^{2} = 0.98 \hfill &\quad {\text{for BCPE}}, \hfill \\ {\text{I}}\left( {\upmu {\text{A}}} \right) = 13.99\upupsilon ^{1/2} - 23.46; \hfill &\quad {\text{r}}^{2} = 0.97 \hfill &\quad {\text{for PTCPE}}, \hfill \\ \end{array}$$

The excellent usage of CPE for adsorbing particles on its active surface during the electrode reaction is reflected in a linearity amongst log I versus log ν with a slope of 0.62 (Fig. 4C), which denotes an adsorption-controlled mechanism. Thus, it is a mixed diffusion-adsorption mechanism overall. These outcomes are in line with the literature on the AM’s transport characteristics at other modified electrodes^46^.

The kinetic factors are generated using Laviron model (Eq. 1) from Fig. 4D, which depicts a linear relationship amongst the peak potential and the logarithm of the scan rate represented as following^47^:

1 $${\text{E}}_{{{\text{pa}}}} = {\text{E}}^{0} + 2.30\,{\text{RT}}/\left[ {\left( {1 - \upalpha } \right){\text{nF}}} \right] \times \log\upnu$$

1 $${\text{E}}_{{{\text{pa}}}} ({\text{V}}) = 1.06 + 0.20\log\upnu \left( {\text{V/s}} \right)\quad \left( {{\text{r}}^{2} = 0.96} \right)$$

Where α is the electron-transfer factor, which for irreversible processes ranges between 0.4 and 0.6^48^ and n is the number of electrons transferred in the electro-oxidation reaction, which is estimated from Eq. (1) to be roughly 1.0 for AM.

Applying the Randles-Sevcik equation (Eq. 2), the active surface area of PTCPE was calculated using the CV technique in 1.0 mM K_4_Fe(CN)_6_ and 0.1 M KCl as an electrolyte^49^:

2 $${\text{I}}_{{{\text{pa}}}} = \left( {2.69 \times 10^{5} } \right){\text{n}}^{3/2} {\text{ACD}}^{1/2}\upnu ^{1/2}$$

Where I_pa_ is the anodic peak current (A), n is the number of transferred electrons through the redox process and *n* = 1.0, A is the electrode’s electro-active area (cm^2^), C is the concentration of K_4_Fe(CN)_6_ (mol/cm^3^), D is the diffusion coefficient (cm^2^/s) equal to 7.6 × 10^−6^, and ν is the scan rate (V/s). The electro-active area is determined to be 0.05 cm^2^ for the BCPE, and 0.15 cm^2^ for the PTCPE.

According to Eq. (2), (2.69 × 10^5^) n^3/2^ACD^1/2^ corresponds to the slope in Fig. 4B and by substituting with the estimated area; both BCPE and PTCPE are thought to have diffusion coefficients of 51.42 × 10^−3^ and 120.21 × 10^−3^ cm^2^/s respectively. Thus, the addition of PT to the sensor dramatically improved the AM molecules’ diffusion through the electrolyte and enlarged the active surface area by nearly a factor of 3.0.

### Influence of accumulation time

CVs for 1.0 mM AM in PBS (0.1 M, pH 7.4) were performed over various time intervals to investigate the response of PTCPE (Supplementary Fig. S2). The anodic peak currents enlarged when the sensor’s immersion time was increased until reaching a plateau after nearly 140.0 min, that may be attributable to the obstructing of the active sites along the electrode’s surface by the molecules of the oxidation products, so no more sensing can occur. This time was considered to be the optimal time for the electrode stability.

### Calibration curve study

**Fig. 5:**
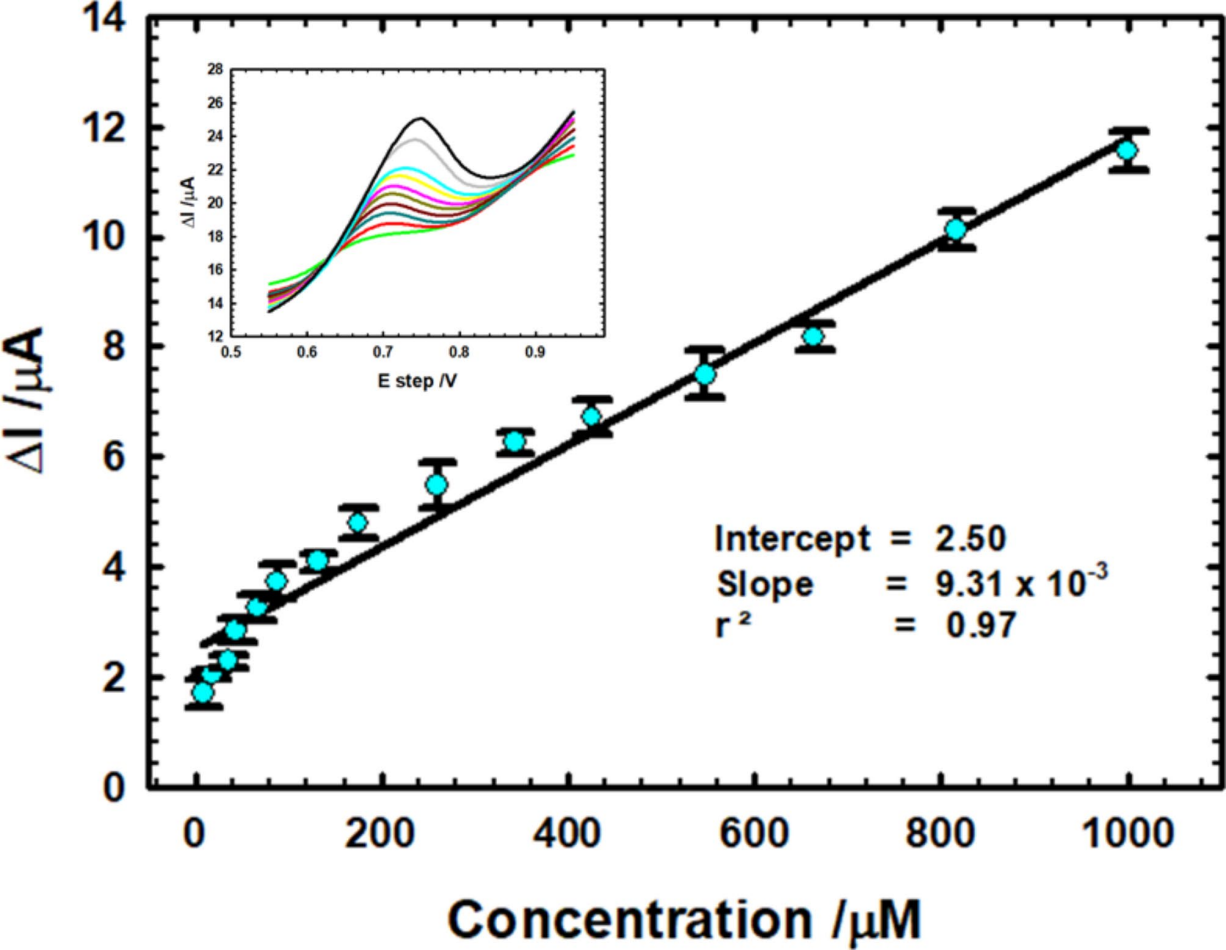
The calibration diagram of AM in PBS (pH 7.4) using PTCPE. Inset: the equivalent curves for rising AM concentrations using SWV at scan rate 0.01 V/s.

Linearity among anodic peak current and multiple AM concentrations (Fig. 5) confirms the sensitivity of PTCPE for electro-detection of AM and it can be expressed by the linear equation:

$${\text{I}}\left( {\upmu {\text{A}}} \right) = 2.50 + 9.31 \times 10^{ - 3} \,{\text{C}}\;\left( {\upmu {\text{M}}} \right)\quad \left( {{\text{r}}^{2} = 0.97} \right)$$

The associated SWV curves for rising AM concentration from 8.88 µM up to 1000.00 µM in PBS (0.1 M, pH 7.4) and scan rate 0.01 V/s using PTCPE are shown in the inset of Fig. 5. The following Eqs. (3 and 4) are used to compute the limit of detection (LOD) and limit of quantification (LOQ)^50,51^:

3 $${\text{LOD}} = 3\,{\text{s/m}}$$

4 $${\text{LOQ}} = 10\,{\text{s/m}}$$

and it was discovered that they were 0.32 and 1.07 µM, respectively, proving the electrode’s sensitivity. A relative standard deviation (RSD) of 1.43% was obtained after repeating the measurements five times under the identical circumstances to test the suggested electrode’s repeatability.

Table 2 provides a comparative analysis of some of the formerly reported techniques for AM detection. Nevertheless, these methods require the use of expensive or toxic chemicals, in addition to being more difficult to fabricate than the edited electrode presented here. Thus, this method demonstrated its dependability and sensitivity for AM detection with a relatively low detection limit and great selectivity.

**Table 2:** PTCPE against other analytical and electrochemical methods in the literature.

Method	Electrode’s material	Linear range (µM)	LOD (µM)	References
Spectrophotometric analysis	–	6.68–66.76	1.60	^14^
Colorimetric sensing	–	0.2–20.0	0.05	^15^
HPLC–ELSD^a^	–	67.9–679.9	9.01	^52^
RP-HPLC^b^	–	33.38–367.16	16.7	^13^
DPV^c^	MIP/SPCE^d^	0.5–10.0	0.08	^25^
SWV	Hg(Ag)FE^e^	6.42–31.11	1.92	^28^
DPV	MIP/ABP^f^	0.1–20.0	0.01	^26^
DPV	FSCPE^g^	44.0–1000.0	11.0	^43^
SWV	PTCPE	8.88–1000.0	0.32	This work

^a^HPLC–ELSD: high performance liquid chromatography coupled with an evaporative light scattering detector.

^b^RP-HPLC: reversed-phase-high performance liquid chromatography.

^c^DPV: differential pulse voltammetry.

^d^MIP/SPCE: molecularly imprinted polymer on a screen-printed carbon electrode.

^e^Hg(Ag)FE: silver-amalgam film electrode.

^f^MIP/ABP: molecularly imprinted polymer/acetylene black-mod carbon paste.

^g^FSCPE: fumed silica carbon paste electrode.

### Commercial samples study

The sensor’s feasibility was assessed by detecting AM in pharmaceutical samples using SWV, by spiking the samples with standard AM concentrations applying a standard addition method. Supplementary Table S1 displays the results, which confirm that the modified electrode can accurately measure AM in pharmaceutical samples with recoveries of 99.03–103.22% and RSDs of 1.0–3.4% for all samples. To evaluate the level of AM in the real samples, each measurement was performed with an average of five replicate trials.

### PTCPE’s long-term stability, repeatability, and interference study

Supplementary Table S2 provides an illustration of how different interfering substances affect PTCPE’s ability to detect AM. This was accomplished by loading equal and double amounts of several substances, such as urea, sucrose, glucose, starch, and paracetamol, into a fixed concentration of AM (600.0 µM). To confirm the selectivity of the suggested technique against AM, it was evaluated using the same experimental settings with other antibiotics and structurally related drugs such as erythromycin, clarithromycin, ciprofloxacin, cefixime, and cephalexin.

As a recommended protocol for handling COVID-19 patients, paracetamol is commonly used with AM, so it is essential to examine the PTCPE’s selectivity for it. The sensor was competent for detecting both of them at distinct peak potentials of 0.36 V and 0.80 V for paracetamol and AM, respectively (Supplementary Fig. S3) without altering the sensor’s response to AM, demonstrating the sensor’s great selectivity.

Five sequent voltammetric determinations for 50.0 µM of AM with an RSD of 1.5% were performed to validate the reproducibility of PTCPE in terms of RSD, guaranteeing the precision of the electrode under investigation.

The PTCPE was kept at room temperature for 12.0 days to examine its long-term stability. The sensor was then subjected to a single voltammetric measurement for AM, which revealed a current response of 97.0% of the value recorded right after fresh fabrication, indicating good electrode storage consistency.

## Conclusion

The electrochemical detection of AM was achieved here using the novel and simple PTCP sensor utilizing SWV technique. Several electrochemical techniques were first used to optimize the experimental conditions (such as pH and potential scan rate) leading to the best sensitivity for AM detection. AM oxidation occurred under mixed adsorption-diffusion control mechanism. The proposed approach was easy to use, inexpensive and sensitive enough to detect AM in medicinal capsules under physiological circumstances with acceptable recoveries ranging from 99.03 to 103.22% and RSDs of 1.0–3.4% for all samples, without noticeable interference of any combining substrates. Comparison with some other analytical and electrochemical techniques from literature confirms the good sensitivity of the proposed sensor, with a wide linear range of 8.88–1000.00 µM of AM and a LOD of 0.32 µM.

## Electronic supplementary material

Below is the link to the electronic supplementary material.

Supplementary Material 1

## Data Availability

The datasets used and/or analysed during the current study available from the corresponding author on reasonable request.
