# Supplementary material for: Electro-polymerization of modified carbon paste sensor for detecting azithromycin
Source: Sci Rep. 2025 Jan 6;15:980. doi: 10.1038/s41598-024-79614-6 (PMC11704252; doi:10.1038/s41598-024-79614-6)
Supplement: Supplementary file 1 — Supplementary Material 1 [file 41598_2024_79614_MOESM1_ESM.doc]

Supplementary data

**Electro-polymerization of modified carbon paste sensor for detecting Azithromycin**

**Salma Mamdouh, M. Shehata, A.M. Fekry and M.A.Ameer^*^**

Chemistry Department, Faculty of Science, Cairo University, Giza 12613, Egypt

^*^Corresponding author: Tel: +202-01001675085,

E-Mail: [mameer@Sci.cu.edu.eg/](mailto:mameer@Sci.cu.edu.eg/) [mameer_eg@yahoo.com/](mailto:mameer_eg@yahoo.com/) mameereg@gmail.com

- **Compounds Structures & Surface Characterization**


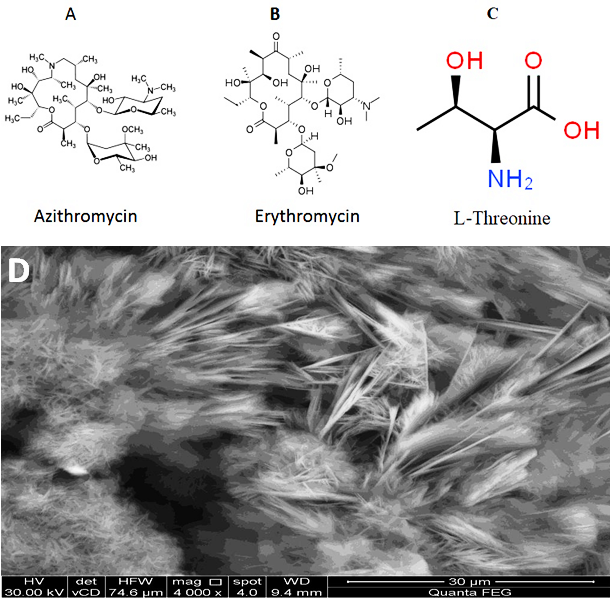


**Fig.S1: Structure of Azithromycin (A), Erythromycin (B) and L-Threonine (C).**

**(D) SEM image of PT on CPE.**


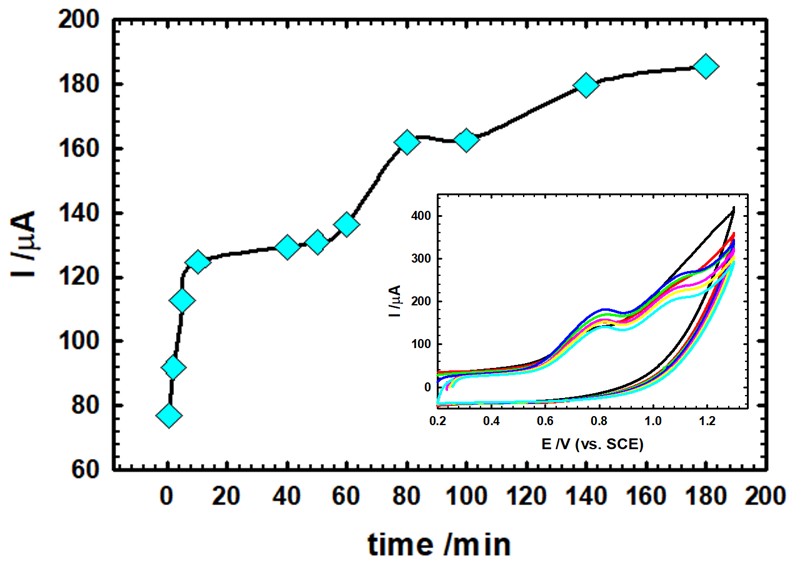


**Fig.S2: Changes in the anodic peak current (Ipa) measured using PTCPE in pH 7.4 PBS (0.1 M) containing 1.0 mM AM for consecutive analyses at increasing times (1.0 – 180.0 minutes). Inset: the equivalent CVs.**


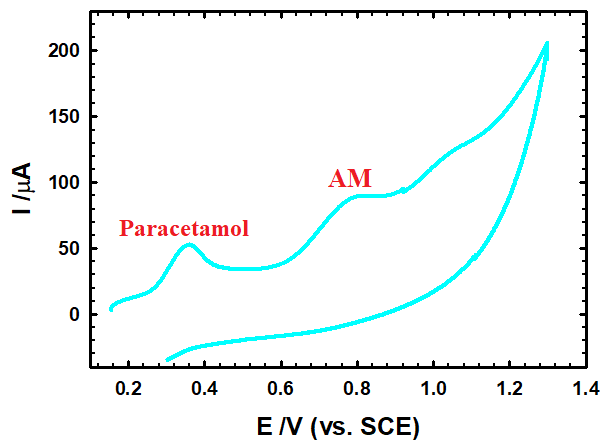


**Fig.S3: CV for PTCPE selectivity towards 600.0 μM of AM in the presence of paracetamol in pH 7.4 PBS** **with scan rate 0.05 V/s.**

**Table S1. Recoveries of AM in pharmaceutical samples at pH 7.4. RSD (%) was estimated for the average of five trials.**

| AM added  (µM) | Expected  (µM) | Found  (µM) | Recovery  (%) | RSD  (%) |
| --- | --- | --- | --- | --- |
| 0 | - | 114.75 | - | 3.4 |
| 4.44 | 119.19 | 118.03 | 99.03 | 1.0 |
| 8.88 | 123.63 | 127.61 | 103.22 | 2.3 |
| 17.75 | 132.50 | 131.35 | 99.13 | 2.1 |
| 35.43 | 150.18 | 149.38 | 99.47 | 1.8 |
| 44.25 | 159.0 | 159.16 | 100.10 | 1.2 |

**Table S2. The impact of interfering materials on sensing of AM by PTCPE.**

| **Interfering material** | **Relative sensor response (%)^*^** | |
| --- | --- | --- |
|  | 1:1 | 1:2 |
| Glucose | 98.4 | 97.1 |
| Sucrose | 97.2 | 96.8 |
| Starch | 98.0 | 97.0 |
| Urea | 99.5 | 99.3 |
| Paracetamol | 98.7 | 98.1 |
| Erythromycin, ciprofloxacin, cefixime, cephalexin and clarithromycin | 97.9 | 96.3 |

* Average of five measurements
